# Research status and hotspots of hypothermia and human diseases: a bibliometric analysis

**DOI:** 10.3389/fmed.2025.1518173

**Published:** 2025-02-21

**Authors:** Wei-Xuan Li, Na-Na Han, Qian-Yu Ji, Xue-Tong Dong, Chao-Long Lu, Song-Jun Wang

**Affiliations:** ^1^Undergraduate of College of Forensic Medicine, Hebei Medical University, Shijiazhuang, China; ^2^Undergraduate of College of Basic Medicine, Hebei Medical University, Shijiazhuang, China; ^3^Hebei Key Laboratory of Forensic Medicine, Research Unit of Digestive Tract Microecosystem Pharmacology and Toxicology, Collaborative Innovation Center of Forensic Medical Molecular Identification, College of Forensic Medicine, Chinese Academy of Medical Sciences, Hebei Medical University, Shijiazhuang, China

**Keywords:** bibliometric analysis, hypothermia, human disease, injury, CiteSpace, VOSviewer

## Abstract

**Background:**

Hypothermia has been strongly associated with human diseases; it affects life safety. Therapeutic hypothermia generates good results for certain diseases, without serious complications. In clinical practice, research on the treatment of hypothermia and severe hypothermia-induced diseases have achieved fruitful results. However, no bibliometric analysis has been conducted. In this study, we explored the research status and hotspots of hypothermia and human diseases by conducting a bibliometric analysis.

**Methods:**

Articles on hypothermia and human diseases were collected from the Web of Science Core Collection. From 1 January 2005 to 31 August 2024, A total of 1,553 articles were retrieved. After excluding irrelevant articles, 706 articles were analyzed.

**Results:**

The United States and China published the maximum number of research articles on hypothermia and human diseases. Among institutes, Johns Hopkins University and Harvard University published the maximum number of research articles. Scholars, including Ishikawa Takaki, Maeda Hitoshi, and Michiue Tomomi, constituted a highly productive group of authors. The journal, Therapeutic Hypothermia and Temperature Management published the highest number of articles, and Nature Reviews Drug Discovery had the highest impact factor. Cluster analysis of all keywords primarily focused on the following research directions: (i) hypothermia-related injury, (ii) hypothermia treatment, and (iii) the mechanism underlying hypothermia.

**Conclusion:**

This bibliometric study comprehensively summarizes the impact of hypothermia on human diseases and the research overview of the use of moderate hypothermia for treatment. This paper clarifies the research status, frontiers and hotspots, and also puts forward new insights for hypothermia research: strengthen research cooperation to improve the depth of research, increase support for areas with insufficient medical conditions; in the future, single-cell multiomics technology will be used to explore cell types sensitive to different low temperatures and corresponding molecular mechanisms; non-coding RNA regulation will be used to achieve precision treatment of hypothermia diseases; Organoids will be an important object of hypothermia research. These research insights can provide reference for researchers.

## 1 Introduction

Under normal physiological conditions, the core temperature is maintained at 37 ± 0.5°C in humans. Hypothermia is defined as a core temperature < 35°C. Hypothermia can be divided into mild (core temperature 32–35°C), moderate (core temperature 28–32°C), and profound (core temperature < 28°C) hypothermia (1). Because of climate changes, the incidence of extreme cold weather has increased, substantially increasing cases of frostbite and freezing death (2). Hypothermia-induced diseases in human have attracted widespread research attention (3). In clinical practice, therapeutic hypothermia has been widely used in the treatment of neonatal hypoxic-ischemic encephalopathy and other diseases; it has achieved a remarkable curative effect (4). These two seemingly contradictory but strongly related topics have aroused research interest.

Studies have shown that therapeutic hypothermia focuses on the treatment of brain diseases, such as ischemia-reperfusion injury of the brain (5), neonatal ischemic-hypoxic encephalopathy (6) and neurodegenerative diseases (7). Severe hypothermia can cause systemic damage, such as acute kidney injury (8), lung inflammation (9) and liver injury (10). Through bibliometric analysis, we can effectively understand the research progress and dynamics in the fields of therapeutic hypothermia and severe hypothermia, as well as the differences and connections between these two fields.

Bibliometric analysis effectively assesses general trends in research activities and analyzes linkages between relevant research institutes (11). This method is commonly used to evaluate the credibility, quality, and influence of academic work by quantitatively analyzing the contour distribution, relationship, and clustering of research fields. Additionally, it objectively assesses the development status of a field and reflects the development of disciplines (12). Bibliometric analysis analyzes the development trend of literature, discipline frontier, research hotspots, author cooperation, and influencing factors. It provides important information for strategic planning and resource allocation of research directions (13). With the increasing volume of research literature and the importance of their influence, bibliometric analysis will play an important role in research.

In-depth research on the protective mechanism underlying therapeutic hypothermia and the injury mechanism underlying severe hypothermia have obtained fruitful results. However, no bibliometric analysis has been conducted. In this study, we aimed to use CiteSpace and VOSviewer to conduct a bibliometric analysis to comprehensively, scientifically, and intuitively present the research history, trends, and hotspots in the form of charts. Our findings will provide a valuable reference for future academic development trends.

## 2 Materials and methods

### 2.1 Data source and literature search strategy

Web of Science is a large global, comprehensive, multidisciplinary, and high-impact academic information repository. It comprises articles in natural science, biomedicine, and other fields, thus guaranteeing our high-quality research analysis (13, 14). Articles on hypothermia and human diseases were retrieved from the Web of Science Core Collection (WoSCC) from 1 January 2005, to 31 August 2024. The following search terms were used: [TS = (hypothermia) OR TS = (low temperature) OR TS = (cold stress) OR TS = (cold exposure)] AND TS = (disease) AND TS = (injury). A total of 1,553 articles were retrieved. In order to ensure the quality and reliability of the data, we only included the original research papers and review articles that have been published. The conclusions and data of conference papers and other types of articles may not have been fully verified because they have not been peer reviewed and the content may change. Citing these literatures may affect the accuracy and reliability of our research conclusions, so they were not included in this study. During the literature screening process, we also excluded articles that are not related to the topic of this study and duplicate publications. Through this series of rigorous screening steps, we finally included and analyzed 706 high-quality articles. They were saved as plain text files and exported as full records and cited references.

### 2.2 Bibliometric analysis software

**FIGURE 1:**
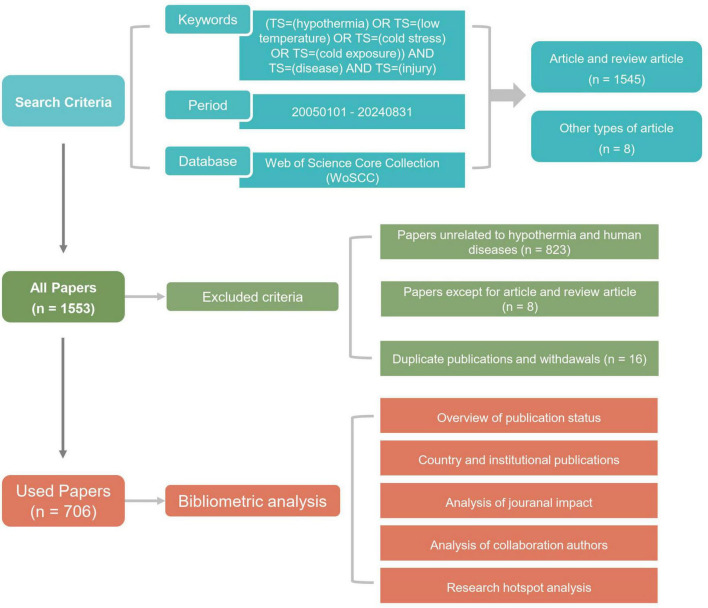
Flowchart of the study.

This study used CiteSpace 6.4.R1, VOSviewer 1.6.20, R version 4.4.0 and Microsoft Office Excel 2019 as the software for bibliometric analysis. CiteSpace is a document visualization analysis software widely used for bibliometric analysis and data visualization (15, 16). VOSviewer has strong graphics processing capabilities and processes large-scale data (17). The number of publications were analyzed using the linear growth function in Excel; VOSviewer was used for analyzing and visualizing collaboration among countries, institutes, and authors, as well as keyword co-occurrence and overlay analysis. CiteSpace was used to analyze the double-graph overlay of journals and keyword timelines and to identify the burst keywords (Figure 1).

## 3 Results

This section may be divided by subheadings. It should provide a concise and precise description of the experimental results, their interpretation, as well as the experimental conclusions that can be drawn.

### 3.1 Overview of publication status

**FIGURE 2:**
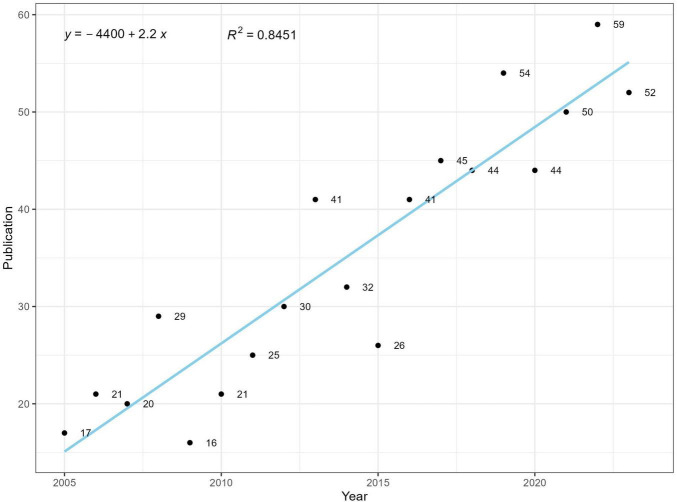
Number of publications per year.

The number of publications can reflect the pace and trend of research (17). An enumeration analysis of annual publications on hypothermia and human diseases since 2005 (Figure 2) demonstrated a gradual increase from 17 to 52 (2005–2023). The number of publications has declined in some years; nonetheless, the overall trend is steadily increasing. We assessed the publication versus year using a linear growth function; *R*^2^ = 0.8451 indicated a strong correlation, suggesting that articles on hypothermia and human diseases show significant growth and development. Additionally, the correlation between hypothermia and human diseases has attracted widespread attention from researchers.

### 3.2 Country-wise contributions

**FIGURE 3:**
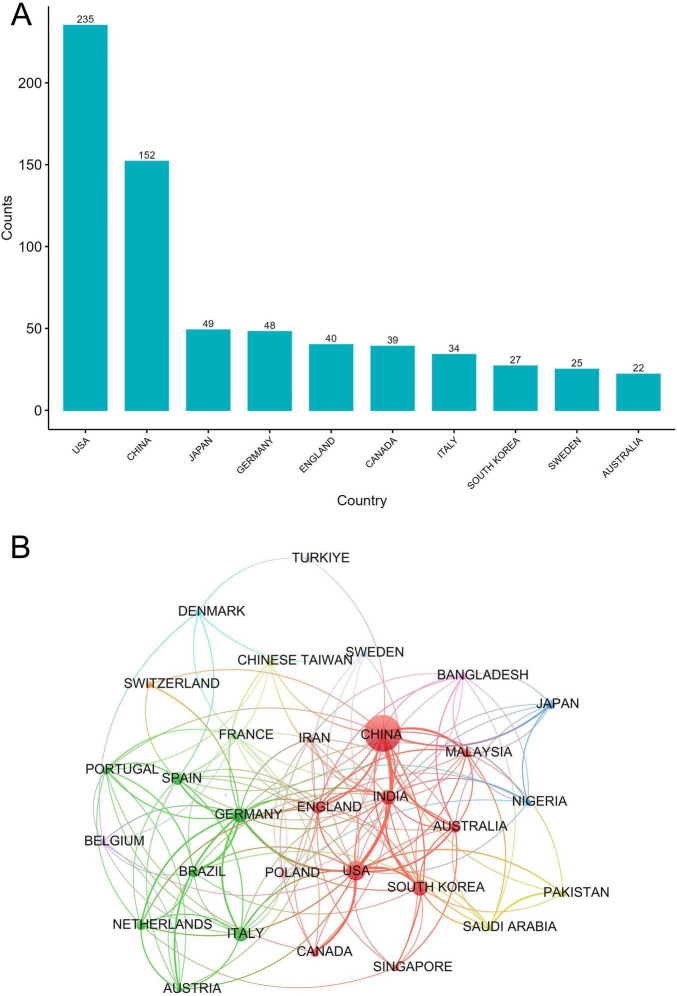
Country-wise contributions. **(A)** National publication counts. **(B)** Mapping collaborative networks among countries/regions.

We analyzed the number of research articles published by countries to investigate the countries/regions that have contributed substantially to hypothermia and human diseases. The United States published the maximum number of research articles on hypothermia and human diseases (235), followed by China (152), Japan (49), Germany (48), England (40), and Canada (39) (Figure 3A). The cooperation among countries was visualized using VOSviewer (Figure 3B). The United States demonstrated the closest cooperation with China, followed by Canada. Strong cooperation and contacts were visualized among other countries, suggesting close academic ties and frequent academic interactions. Good academic cooperation will promote rapid research development.

### 3.3 Institute-wise contributions

**FIGURE 4:**
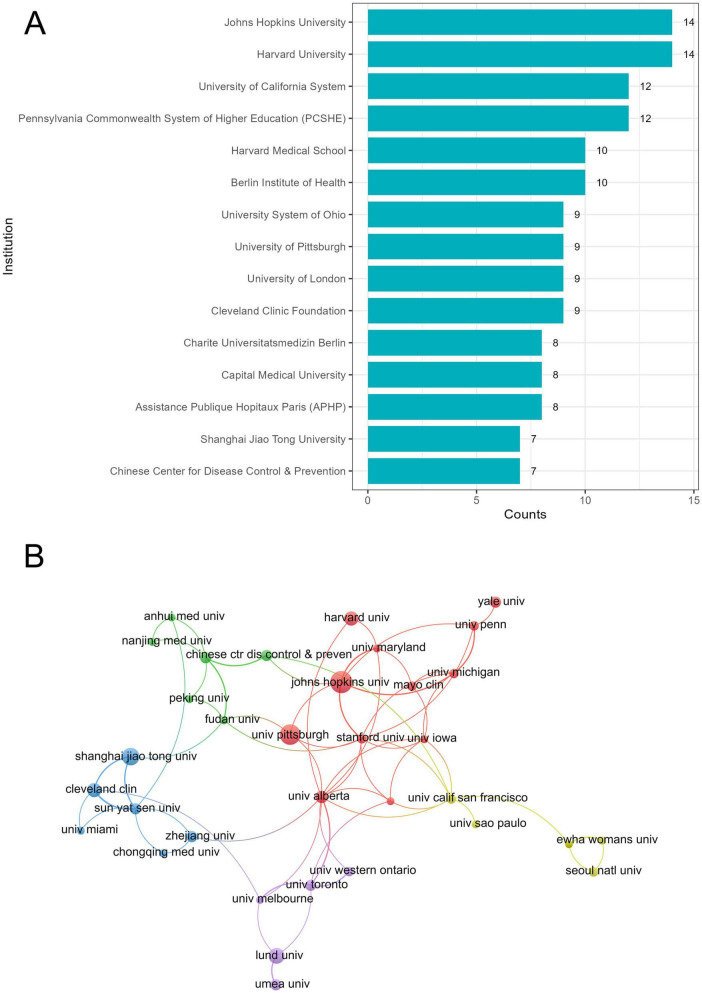
Institute-wise contributions. **(A)** Number of institute publications. **(B)** Mapping collaborative networks among institutes.

A total of 402 institutes research hypothermia and human diseases worldwide. Johns Hopkins University and Harvard University (14) published the maximum number of research articles, followed by the University of California System and Pennsylvania Commonwealth System of Higher Education (12) (Figure 4A). The cooperation among institutes can reflect the research activity (Figure 4B). The strongest cooperation was observed between the University of Pittsburgh and Johns Hopkins University, followed by that between the China Medical University and Osaka City University and Cleveland Clinic Foundation and Shanghai Jiao Tong University.

### 3.4 Author-wise contributions

**FIGURE 5:**
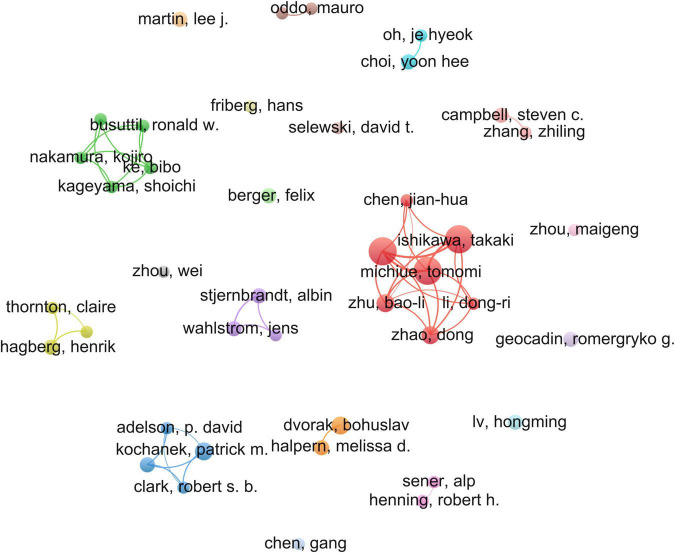
Mapping collaborative networks among authors.

A total of 632 authors had written research articles on hypothermia and human diseases (Figure 5). Among them, Ishikawa Takaki, Maeda Hitoshi, Michiue Tomomi, Zhao Dong, and Zhu Bao-li constituted the first echelon of high-yield authors, followed by Halpern Melissa D., Dvorak Bohuslav, among others. Additionally, researchers from China developed a novel and powerful research network on hypothermia and human diseases.

### 3.5 Analyzing publication quantity and journal impact

**FIGURE 6:**
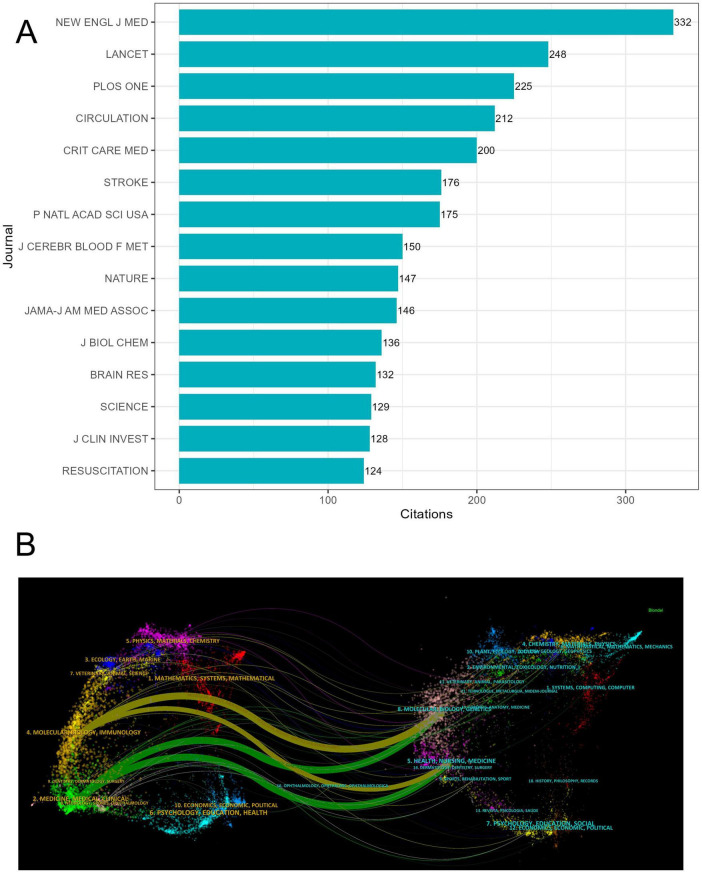
Journal-wise contributions. **(A)** Number of citations. **(B)** Double-graph overlay analysis of citing and cited journals.

The leading 20 journals have been listed by the latest impact factor (IF) (Table 1). Nature Reviews Drug Discovery had the highest impact factor (IF = 122.7), followed by Lancet (IF = 98.4). All leading 20 journals with impact factor rankings were in the first quartile (Q1) of the Journal Citation Reports. Among them, the United States and the United Kingdom published the highest number of journals (eight journals each). Gastroenterology, Lancet Public Health, European Urology, and Science Translational Medicine published the most papers, with two. Therapeutic Hypothermia and Temperature Management published the highest number of articles (13), followed by PLoS One (11). The New England Journal of Medicine was the most cited journal (332 citations), followed by the Lancet (248 citations) (Figure 6A). The quality of articles suggested that research on hypothermia and human diseases has been recognized and commended by academic circles.

**TABLE 1:** Journals with the latest impact factor.

Rank	Source	IF	Country/Region	JCR-c	Article
1	Nature Reviews Drug Discovery	122.7	England	Q1	1
2	Lancet	98.4	England	Q1	1
3	New England Journal of Medicine	96.2	United States	Q1	1
4	Lancet Neurology	46.5	England	Q1	1
5	European Heart Journal	37.6	England	Q1	1
6	Nature Reviews Neurology	28.2	United States	Q1	1
7	Intensive Care Medicine	27.1	United States	Q1	1
8	Gastroenterology	25.7	United States	Q1	2
9	Lancet Public Health	25.4	United Kingdom	Q1	2
10	European Urology	25.3	Netherlands	Q1	2
11	Journal of the American College of Cardiology	21.7	United States	Q1	1
12	Blood	21.0	United States	Q1	1
13	Jama Neurology	20.4	United States	Q1	1
14	Bioactive Materials	18.0	China	Q1	1
15	European Respiratory Journal	16.6	England	Q1	1
16	Science Translational Medicine	15.8	United States	Q1	2
17	Nature Communications	14.7	England	Q1	1
18	Cell Death and Differentiation	13.7	England	Q1	1
19	Chemical Engineering Journal	13.3	Switzerland	Q1	1
20	Drugs	13.0	New Zealand	Q1	1

Double-graph overlay analysis of the citing and cited journals facilitated measuring and visualizing the data to elucidate the flow process of the knowledge system (Figure 6B). Four key citation paths were identified, and the fields of citing journals focused on “Molecular, Biology, Immunology” and “Medicine, Medical, Clinical.” The fields cited focused on “Molecular, Biology, Genetics” and “Health, Nursing, Medicine.” Thus, research on hypothermia and human diseases, encompassing both basic and practical application research, focuses on pathological mechanisms and clinical treatment.

### 3.6 Research hotspot analysis

#### 3.6.1 Keyword frequency and co-occurrence analysis

**FIGURE 7:**
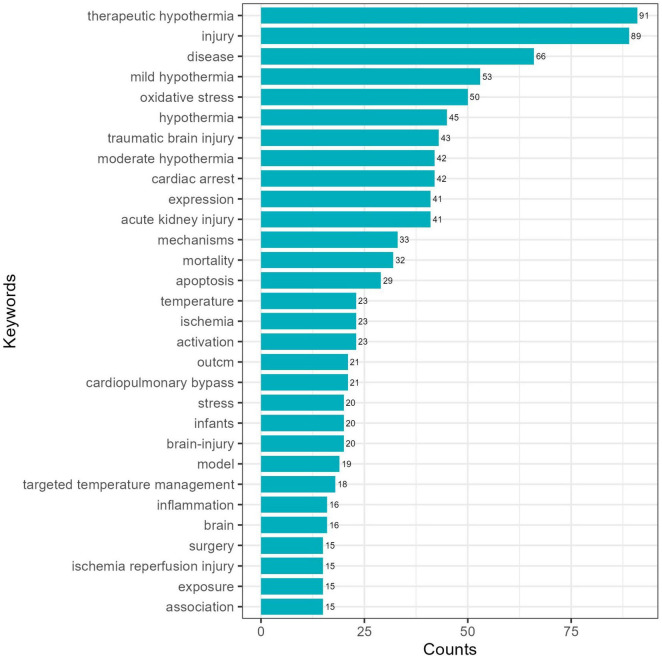
List of the 30 most frequently used keywords.

**FIGURE 8:**
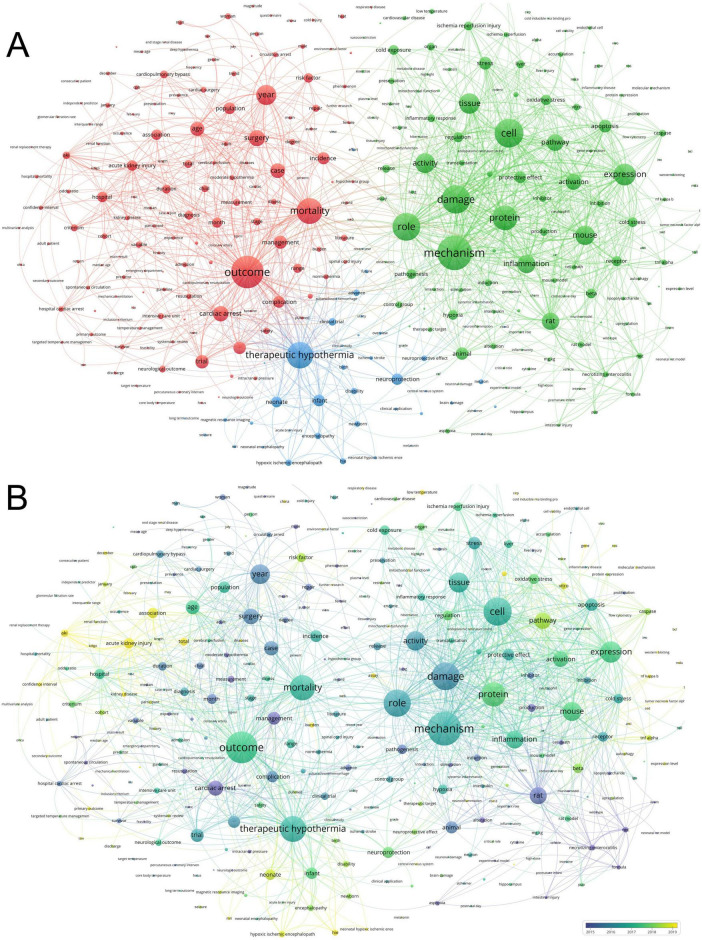
Keyword analysis. **(A)** Keyword co-occurrence network. **(B)** Time-overlapping co-occurrence network analysis of keywords.

Keywords offer a refined presentation of academic contributions, reflecting the research focus and direction. Based on the frequency (Figure 7), “therapeutic hypothermia” was the most frequent keyword with (*N* = 91), followed by “injury” (*N* = 89), “disease” (*N* = 66), “mild hypothermia” (*N* = 53), and “oxidative stress” (*N* = 50). The co-occurrence analysis of keywords facilitates rapidly identifying the research hotspots. This study comprises 496 keywords, of which 11 appear more than 40 times. Cluster analysis of all keywords primarily focused on the following research directions (Figure 8A): (i) hypothermia-related injury comprising keywords, including “outcome,” “cardiac arrest,” “surgery,” “mortality,” “complication,” and so on; (ii) hypothermia treatment comprising keywords, including “therapeutic hypothermia,” “hypoxic-ischemic encephalopathy,” “spinal cord injury,” “ischemic stroke,” and so on; and (iii) the mechanism underlying hypothermia comprising keywords, including “mechanism,” “protein,” “damage,” “protective effect,” “expression,” and so on. The keyword overlay network spectrum illustrates the gradual trend of keyword changes. Keywords, such as “pathway,” “caspase,” “TNF alpha,” and “neonate” were frequent and may be identified as research hotspots in the future (Figure 8B).

#### 3.6.2 Keyword timeline chart analysis

**FIGURE 9:**
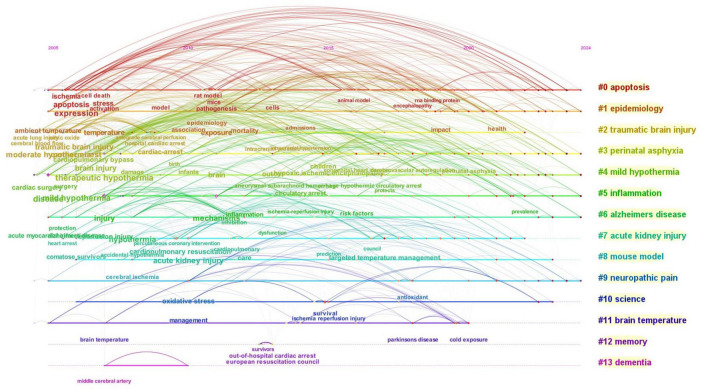
Keyword timeline map.

The keyword timeline chart reflects the correlation of each keyword cluster and the span of publication time corresponding to each cluster. It enables analyzing the research topic, starting time, and development trend reflected by each cluster. According to the start time and duration of each keyword cluster research, the topics of hypothermia and human diseases were divided into four categories (Figure 9) as follows: (1) research topics with an early start and long duration, including five keyword clusters, namely # 0 apoptosis, # 1 epidemiology, # 4 mild hypothermia, # 6 Alzheimer’s disease, and # 9 neuropathic pain. The key research contents included cerebral ischemia, apoptosis, RNA binding protein, encephalopathy, and therapeutic hypothermia, among others; (2) research topics with an early start and short duration, including two keyword clusters, namely # 2 traumatic brain injury and # 11 brain temperature. It primarily focused on the research of ambient temperature, mortality, exposure, Parkinson’s disease, and cold exposure, among others; (3) research topics with a delayed start and long duration, including two keyword clusters, namely # 3 perinatal asphyxia and # 5 inflammation. It focused on moderate hypothermia, cardiac arrest, and circulatory arrest, among others; and (4) research topics with a delayed start and short duration, including five keyword clusters, namely # 7 acute kidney injury, # 8 mouse model, # 10 science, # 12 memory, and # 13 dementia. The key research contents included “comatose survivors,” “oxidative stress,” “antioxidant,” “middle cerebral artery,” “survivors,” “out-of-hospital cardiac arrest,” and so on.

#### 3.6.3 Keyword burst analysis

**FIGURE 10:**
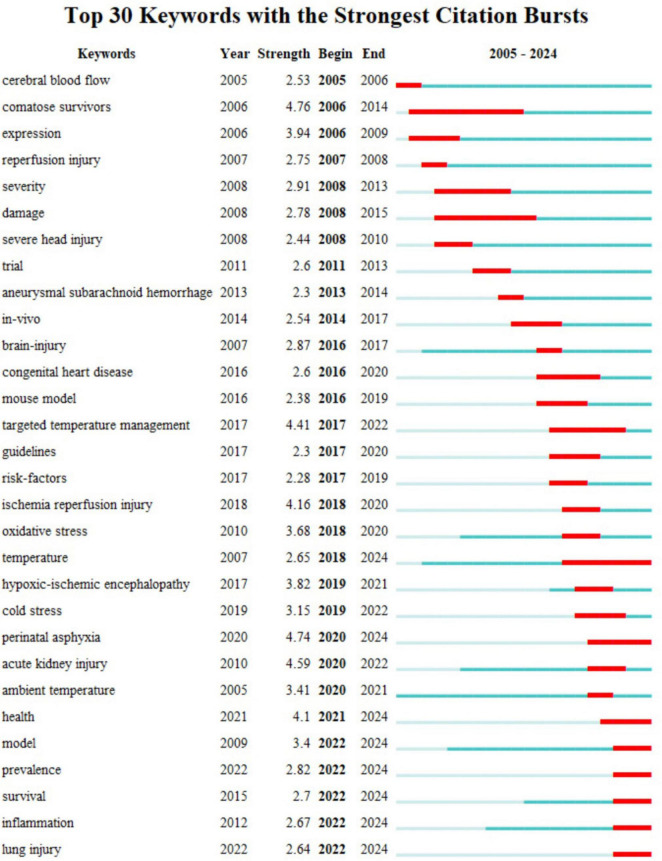
Thirty keywords with the strongest citation bursts.

Keyword burst suggests that the number of citations of a research article is significantly higher than the normal number of citations and lasts for at least 2 years (16). The keyword “comatose survivors” had the strongest citation burst (4.76), followed by “perinatal asphyxia” (4.74), “acute kidney injury” (4.59), “targeted temperature management” (4.41), “ischemia-reperfusion injury” (4.16), and “health” (4.1). According to the start time of appearance, “cerebral blood flow,” “comatose survivors,” “expression,” and “reperfusion injury” appeared earlier and were the chief concerns of early researchers. Model, prevalence, survival, inflammation, and lung injury were the research frontiers of hypothermia and human diseases, visible in the outbreak period (Figure 10). Thus, the mentioned keywords are possible research hotspots in the future.

## 4 Discussion

### 4.1 Basic information

In this study, we conducted a bibliometric analysis to analyze the studies on hypothermia and human diseases from 2005 to 2024. Since 2005, hypothermia and human diseases research has displayed a rapid growth trend. Particularly in 2022, 59 articles were published annually, indicating that research hotspots associated with hypothermia and human diseases have increasingly attracted attention. The increasing incidence of extreme cold weather has contributed to the widespread research attention on human diseases (18–20). In clinical practice, hypothermia has been recognized as a treatment mode. These factors have contributed to the rapid development of this field.

This study included 706 research articles published across 76 countries. Of these countries, the United States and China published the highest number of articles. Additionally, among the countries with the highest frequency of cooperation, the United States plays a central role in international cooperation. Therefore, the United States holds a leading position in hypothermia and human disease research and has substantially contributed to this field. This finding may be attributed to the high national economic condition and considerable medical investment in the United States. Moreover, hypothermia treatment requires advanced intensive care support, which may be unavailable in low- and middle-income countries (21). Therefore, strengthening exchanges and cooperation among research institutions in developed countries to improve their technical level and reduce their dependence on expensive instruments and equipment, while strengthening assistance to countries with insufficient medical conditions, may be a key way to solve the problem.

Of the 402 institutes, 13 of the leading 30 institutes are located in the United States, consistent with the country-wise distribution of published articles. China ranks second in the number of publications; nonetheless, only six universities were listed in the leading 30 institutes. Japan ranks third in the number of publications, with only one institute in the leading 30. In contrast, the German Berlin Institute of Health ranks sixth, with 10 publications. Thus, the economy and resources limit the research output; however, various institutes are actively seeking international cooperation to improve scientific and technological competitiveness.

Peer-reviewed journals are central to the publication of scholarly work, with core journals publishing important research. Researchers can identify potential journals for submissions based on the number of published articles focusing on hypothermia and human diseases. Therapeutic Hypothermia and Temperature Management has published the highest number of articles. IF is a common indicator that evaluates a journal’s influence. Nature Reviews Drug Discovery has the highest IF (122.7). Q1 journals accounted for all of the leading 20 journals with IF rankings. Additionally, China has substantially contributed to hypothermia and human disease research; nonetheless, Asian publishing houses are underrepresented in the leading 10 journals. This drawback highlights the need to establish and develop internationally recognized journals in Asia.

### 4.2 Research focus and hotspots

Keywords in a research article reflect the author’s academic contributions, guiding the research direction, framing academic topics, and influencing core articles. Keyword frequency statistics, co-occurrence analysis, and cluster analysis can predict upcoming research topics and hotspots. They are central to assisting researchers explore disciplinary shifts and emerging trends. Based on the leading 25 burst keywords and references in CiteSpace software, the following three research fields and corresponding research hotspots were obtained:

Mechanism underlying the protective effect of therapeutic hypothermia: Therapeutic cerebral hypothermia has a therapeutic role in hypoxic brain injury caused by cardiac arrest (22, 23) and neonatal hypoxic-ischemic encephalopathy (24, 25). Its key protective mechanisms include reducing oxygen consumption in the brain and glucose consumption in low-flow areas (26, 27), delaying destructive enzymatic reactions, inhibiting free radical reactions, protecting lipoprotein membrane fluidity, reducing intracellular acidosis, inhibiting biosynthesis, releasing and uptaking of excitatory neurotransmitters, inhibiting nitric oxide production and apoptosis, repairing DNA damage, and reducing inflammatory reactions (28–31). Researchers should select a cooling method that can not only achieve rapid cooling but also minimize tissue damage. For example, researchers have explored the therapeutic effects of cooling methods, such as systemic hypothermia (32), selective hypothermia, intravascular hypothermia, drug-induced hypothermia (33), and artificial hibernation technology (34) through animal experiments and clinical trials. However, there is a lack of systematic and comprehensive research on the mechanism of therapeutic hypothermia, and there is a lack of sufficient single-cell multimics data and regulatory mechanisms. In view of the differences between experimental animals and humans, the use of organoids for therapeutic hypothermia research may be a hot direction in future research.

Mechanism underlying the damaging effects of severe hypothermia: Severe hypothermia poses a threat to life and can cause freezing to death. The damage to the body is mainly caused by the combined effect of low temperature stress and severe hypothermia. The damaging effects include vascular endothelial cell injury, neuronal mitochondrial swelling, rupture, autophagy, and oxidative stress injury (29, 35–38). Moreover, severe hypothermia activates an inflammatory response, causing pyroptosis, necroptosis (39), and neuronal death. Ferroptosis, triggered by severe hypothermia-induced metabolic disorders is implicated in neuronal death (40). Furthermore, chronic cold exposure increases the risk of lung injury by activating inflammation, oxidative stress, and pyroptosis (9). Cold stress can cause liver damage by activating pathways, such as apoptosis, oxidative stress, and pyroptosis (10). Additionally, hypothermia-induced diseases (3) primarily include cardiovascular diseases, chronic respiratory diseases, metabolic diseases, Alzheimer’s disease, osteoporosis (41), acute kidney injury (42, 43), and acute respiratory infection, which indicate important research topics (44, 45). Although severe hypothermia is a multi-organ and multi-system injury to the body, future research will focus on comprehensiveness while still focusing on the most sensitive cell types to severe hypothermia, such as nerve cells and endothelial cells, which may be key targets for clinical treatment.

Mechanism of action underlying hypothermia: Substantial research progress has been made on therapeutic hypothermia, particularly in neonatal hypoxic-ischemic encephalopathy and other diseases. However, its efficacy varies (46, 47), with numerous mechanisms yet to be elucidated (37). Therapeutic hypothermia exerts a protective effect by inhibiting oxidative stress and inflammation. By contrast, severe hypothermia can cause damage through similar events (48). Thus, to enhance its therapeutic efficacy, researchers should identify steps to optimize the protective effect while minimizing damage. Different organs and different cells are sensitive to temperature differently, and different states of cells respond differently to temperature. It is necessary to emphasize the differences in the regulatory mechanisms of cells by temperature in order to achieve precision therapy. Non-coding RNA has been involved in various aspects of regulatory molecular networks. The regulatory mechanism by which non-coding RNA mediates hypothermia has become a research hotspot. For example, hypothermia-induced miR-25-3p exerts osteoporotic effects by inhibiting osteogenic differentiation and autophagic activity (41). The RNA-binding protein RBM3 prevents nitric oxide-induced neuronal apoptosis via miR-143 (49). Therefore, the mentioned regulatory mechanism may gain attention in the future.

Low temperature is closely related to human life. Low temperature can not only affect the immune system and cause colds, but also lead to frostbite and even freezing to death. It can also play a protective role by reducing cell metabolism through moderate low temperature. Therefore, comprehensive and in-depth research on low temperature can guide clinical precision treatment of related diseases caused by low temperature, and can also guide humans to strengthen protective measures against low temperature. At the same time, it can also improve low temperature treatment methods to better serve human health.

This study has some limitations. First, WoSCC contains a wide spectrum of scientific publications. To ensure high-quality findings, our analysis was restricted to this database; however, it did not affect the overall trends. Second, CiteSpace and VOSviewer cannot completely replace systematic reviews, despite their use in bibliometric analysis. Third, newly published high-quality studies may not have been included because of various factors and should be considered in the future. Overall, our study provides a strong foundation for understanding the research topics, hotspots, and development trends in hypothermia and human diseases.

## 5 Conclusion

This bibliometric analysis used visualization software to explore the research history of hypothermia and human diseases since 2005, retrieving 706 articles from the Web of Science. The United States and China are the leading countries regarding published articles, with Johns Hopkins University and Harvard University being the leading institutes. Scholars, including Ishikawa Takaki and Maeda Hitoshi, constitute a highly productive research group. The journal Therapeutic Hypothermia and Temperature Management has published the highest number of articles, whereas Nature Reviews Drug Discovery has the highest IF. Cluster analysis of the keywords elucidated three research directions, namely hypothermia-related injury, hypothermia treatment, and the mechanism underlying hypothermia. These results highlight potential research hotspots and will likely provide valuable insights into future academic development.

## Data Availability

The original contributions presented in this study are included in this article/supplementary material, further inquiries can be directed to the corresponding authors.
